# Autism in Action: Reduced Bodily Connectedness during Social Interactions?

**DOI:** 10.3389/fpsyg.2016.01862

**Published:** 2016-11-23

**Authors:** C. (Lieke) E. Peper, Sija J. van der Wal, Sander Begeer

**Affiliations:** ^1^Department of Human Movement Sciences, MOVE Research Institute Amsterdam, Vrije Universiteit AmsterdamAmsterdam Netherlands; ^2^Section Clinical Developmental Psychology, Vrije Universiteit AmsterdamAmsterdam, Netherlands; ^3^EMGO Institute for Health and Care Research, Vrije Universiteit AmsterdamAmsterdam, Netherlands

**Keywords:** autism, entrainment, interpersonal coordination, dynamics, reciprocity

## Abstract

Autism is a lifelong disorder, defined by deficits in social interactions and flexibility. To date, diagnostic markers for autism primarily include limitations in social behavior and cognition. However, such tests have often shown to be inadequate for individuals with autism who are either more cognitively able or intellectually disabled. The assessment of the social limitations of autism would benefit from new tests that capture the dynamics of social initiative and reciprocity in interaction processes, and that are not dependent on intellectual or verbal skills. New entry points for the development of such assessments may be found in ‘bodily connectedness’, the attunement of bodily movement between two individuals. In typical development, bodily connectedness is related to psychological connectedness, including social skills and relation quality. Limitations in bodily connectedness could be a central mechanism underlying the social impairment in autism. While bodily connectedness can be minutely assessed with advanced techniques, our understanding of these skills in autism is limited. This Perspective provides examples of how the potential relation between bodily connectedness and specific characteristics of autism can be examined using methods from the coordination dynamics approach. Uncovering this relation is particularly important for developing sensitive tools to assess the tendency to initiate social interactions and the dynamics of mutual adjustments during social interactions, as current assessments are not suited to grasp ongoing dynamics and reciprocity in behavior. The outcomes of such research may yield valuable openings for the development of diagnostic markers for autism that can be applied across the lifespan.

## Introduction

Autism Spectrum Disorder (from hereon: autism) is a lifelong impairing disorder, or group of disorders (prevalence >1%), defined by deficits in social communication and interaction, and restrictive, repetitive interests (Lai et al., 2014). While autism can be diagnosed in preschoolers, recent findings indicate that the mean age of diagnosis is much higher, especially for individuals with autism and a normal IQ (around 50%) (Begeer et al., 2013; Lai and Baron-Cohen, 2015). Presumably these individuals can compensate for their autism until the complexity of social interactions at older ages brings their autism to light. Consequently, a remaining challenge is finding objective diagnostic markers that can help detect autism across the lifespan.

Although autism also entails non-social deficits, behavioral diagnostic markers for autism beyond the preschool years primarily focus on deficits in social interactive behavior. These behavioral markers often rely on standardized clinical observations (e.g., Autism Diagnostic Observation Schedule; Lord et al., 2000) or parent reports (Autism Diagnostic Interview Revised, ADI-R; Lord et al., 1994; Lai et al., 2014). A less obvious component of social competence lies within bodily movement during social interactions. In typically developing (TD) individuals, bodily movements become more connected in social settings, resulting in both imitative (Chartrand and Bargh, 1999) and synchronized movements (Bernieri, 1988). This ‘bodily connectedness,’ i.e., attunement of bodily movement between individuals, is related to psychological connectedness, as expressed by social skills and relational quality (Hove and Risen, 2009; Lumsden et al., 2012; Cook, 2016).

Here, we argue that the concept of bodily connectedness provides a unique opportunity to assess subtle features of social interactions in autism. We focus on social initiative and social reciprocity. Currently the assessment of these key diagnostic criteria for autism is hampered by the focus on static stimuli and unidirectional settings, which do not capture the ongoing mutual adaptations in the unfolding interaction (Lai et al., 2014). Even the ADOS (Lord et al., 2000), which does rely on the observation of live dynamic interactions, uses subjective and relatively coarse interpretations and quantifies behavior in a dichotomous way (scoring behavioral features, such as reciprocity, as either *present* or *absent*). Subtler and less subjective measurements with higher index quality would be an important addition to the diagnostic arsenal. The dynamic (i.e., time-varying) nature of interaction processes may be captured using empirical methods aimed at uncovering variations and differentiations in bodily connectedness of individuals with and without autism (Schmidt et al., 2012). The proposed focus on bodily movement fits with previous suggestions that perceptuo-motor impairments may critically affect socio-cognitive functioning in autism (Bhat et al., 2011; von Hofsten and Rosander, 2012; De Jaegher, 2013).

## Social Limitations in Autism

The term autism stems from the Greek ‘autos,’ meaning ‘self.’ An extreme orientation toward the self in autism is reflected in poor initiative and reciprocity during social interactions. These features have been confirmed in a large number of studies (Duffy and Healy, 2011) and are central domains of diagnostic assessments (Lord et al., 2000). Social limitations in autism have been linked to disrupted early imitation and dysfunctions in the so-called mirror neuron system (i.e., brain regions that are active when an individual performs a specific action, but also when he/she observes another person performing that action; Klin et al., 2003). However, the evidence for a defect mirror neurons system is mixed (Hamilton, 2013) and the nature of limitations in imitation remains poorly understood (Vivanti et al., 2014). A general explanation for the impairments in social initiative and reciprocity states that individuals with autism find social interactions less rewarding, because they fail to appreciate their emotional significance. Indeed, abnormal brain functioning in autism suggests impaired sensitivity to social affiliation and reward at a neural level (Dawson et al., 2002).

Poor social initiative and reciprocity are most apparent during spontaneous interactions between individuals. Detailed assessment of these impairments requires measuring the dynamics of ongoing interactions, to capture potential asymmetries in the mutual contributions of interacting individuals. To date, tests for social limitations of individuals with autism focus primarily on isolated elements within this dynamical process. For instance, various instruments are available for testing children’s conceptual understanding of perspective taking (Theory of Mind) or emotions (Yirmiya et al., 1998; Begeer et al., 2008). These tests typically focus on unidirectional interactions (“Do I understand what you think?”), and fail to address the dynamics of ongoing interactions.

An additional problem is that conceptual tests target cognitive skills. Normally intelligent individuals with autism (around 50%; Wingate et al., 2014) rely on cognitive abilities to compensate social limitations. This enables them to disguise these limitations, particularly during conceptual (Scheeren et al., 2013) or standard situations (Begeer et al., 2010), while remaining limited in real-life interactions (Klin et al., 2003). Tests for social behavior are often insensitive for more able individuals with autism, at school age or up (Happe, 1995). For intellectually disabled individuals with autism, it is equally important to develop IQ-independent assessments of their social limitations, as social and intellectual limitations are difficult to disentangle (Tureck and Matson, 2012).

The assessment of the social limitations of autism would benefit from tests that (i) capture the dynamics of social initiative and reciprocity in interaction processes, and (ii) are not susceptible to cognitive compensation, or dependent on intellectual or verbal skills. There is a particular scarcity of measures that assess elementary social limitations during direct social interactions, taking into account who initiates the interaction, how interactants are influenced by social triggers, and to what extent they contribute to the interaction in a balanced manner.

## Bodily Connectedness as Marker for Social Abilities

In TD individuals, matching and synchronization of bodily movements are associated with (psychological) characteristics of the interactants and the quality of their relationship, such as self-esteem (Lumsden et al., 2014), pro-social attitudes (Lumsden et al., 2012), physical attractiveness (Zhao et al., 2015), rapport (Hove and Risen, 2009; Raffard et al., 2015), and perceived social difference (Miles et al., 2011). Moreover, moving in synchrony fosters cooperative abilities (Wiltermuth and Heath, 2009; Valdesolo et al., 2010). As bodily connectedness appears to be stronger between individuals whose movement patterns resemble each other (Słowiński et al., 2016), Cook (2016) argued that such connectedness between individuals with and without autism may be reduced due to a mismatch between their movement patterns, given the atypical patterns observed for autism (Bhat et al., 2011; Gowen and Hamilton, 2013). Hence, bodily connectedness may provide insight into underlying processes of social limitations in autism, potentially inspiring new assessment procedures.

Whereas examinations of interactional synchrony using temporal coding for specific actions or rating scales provide rather coarse-grain indices of synchrony (Schmidt et al., 2012), subtler aspects of how persons attune their movements to each other can be assessed using methods from the coordination dynamics approach (Schmidt et al., 2011, 2012). This approach highlights how on-going, dynamic interaction processes play a defining role in interpersonal coordination (Schmidt et al., 1990, 2011; Issartel et al., 2007; Peper et al., 2013). When two persons perceive each other’s rhythmic movements, the resulting interactions yield attraction toward an interpersonal movement synergy (referred to as ‘entrainment’), both in the presence and absence of instructions regarding coordination of the movements. Stronger entrainment reflects stronger mutual interactions. This focus on interpersonal interactions conveys new potential for assessing specific limitations in autism. Indeed, first applications to dyads involving a person with autism (Marsh et al., 2013; Fitzpatrick et al., 2013, 2016) indicated reduced entrainment, suggesting weakened bodily connectedness.

As outlined below, extending the examination of interpersonal coordination dynamics beyond the level of basic entrainment experiments may provide new tools for assessing social initiative and reciprocity. This requires strategically chosen conditions and methods to delineate the degree to which individuals contribute to the entrainment with the other person. If research along these lines is indeed successful, a next step would be to derive assessment tools suitable for clinical settings.

## Interpersonal Coordination Dynamics as Window into Social Limitations in Autism

### Quantifying Entrainment between Two Persons

Signs of bodily connectedness have been reported for TD individuals when they are engaged in a mutual task, even when the bodily movements are immaterial to the joint task performance [e.g., when solving a cognitive puzzle through verbal interaction (Shockley et al., 2003)]. As the limitations in social reciprocity are a defining criterion for an autism diagnosis, such spontaneous attunement of task-irrelevant movements is expected to be reduced in individuals with autism.

The paradigm developed by Shockley et al. (2003) provides an excellent option for examining this prediction. This paradigm involves two persons standing, each looking at a picture, without seeing the picture the partner is looking at. Through verbal communication they have to discover 10 differences between the two pictures. In control measurements the participants do not interact with one another. Shockley et al. (2003) demonstrated that engagement in this joint task resulted in subtle entrainment features in the postural sway patterns of the two TD partners. The degree of this entrainment may be expected to be smaller in autism–TD dyads than in TD–TD dyads. Given the complexity of the obtained postural sway patterns, detailed analysis of their entrainment requires refined analysis methods, such as Cross Recurrence Quantification Analysis (Shockley et al., 2003).

A more common way to determine movement entrainment is to examine the extent to which the movements of two persons are adapted toward each other during rhythmic movements, as those allow for examination of the degree of synchronization over a large number of movement cycles. In TD–TD dyads, entrainment has thus been determined during instructed mutual coordination (e.g., intentional synchronization; Amazeen et al., 1995; Richardson et al., 2007) but also in the absence of such instructions (Schmidt and O’Brien, 1997; Richardson et al., 2007; Oullier et al., 2008). By examining the phase relation (typically referred to as ‘relative phase’) between the two movement patterns, the occurrence and strength of entrainment can be determined. When no stable coordination pattern is observed (indicating weak interpersonal coupling), temporary attraction to synchronized patterns can be determined based on the distribution of relative phases over a trial or by means of recurrence or coherence measures (Ridderikhoff et al., 2006; Richardson et al., 2007, 2008). For stable coordination patterns, the variability of relative phasing between the moving individuals reflects the strength of connectedness (or: coupling), with lower variability reflecting stronger connectedness (Varlet et al., 2012).

Although autism has scarcely been examined along such lines, autism–TD dyads have been found to show less entrainment than TD–TD dyads (Fitzpatrick et al., 2013, 2016; Marsh et al., 2013), suggesting that autism is indeed associated with reduced bodily connectedness. However, although relative phase measures provide information about the degree of synchronization within a dyad, they do not inform us directly about potential differences in how the two individuals contribute to the entrainment process. Hence, additional manipulations and analyses are required to address social initiative and reciprocity asymmetries in more detail.

### Social Initiative

Individuals with autism typically show reduced social initiative. When prompted, some individuals with autism respond adequately (Shabani et al., 2002), but their limited *spontaneous* social initiative remains poor. A prerequisite for testing reduced initiative in social situations is the absence of prompts, instructions, or other cues to trigger behavior (Backer van Ommeren et al., 2015). Tests that rely on spontaneous skills are more sensitive to autism than tests that provide an opportunity to use cognitive skills (Senju et al., 2009). A focus on involuntary bodily connectedness provides a clear advantage here, as it is difficult to compensate for a lack of uninstructed, subtle attunement of bodily movement.

Given their diminished social initiative, we may expect that bodily connectedness in individuals with autism depends on instructions regarding the interactions with another person. Whereas TD individuals tend to synchronize their movements to those of a partner spontaneously, even without being instructed to do so (Schmidt and O’Brien, 1997; Richardson et al., 2007; Oullier et al., 2008), this spontaneous tendency seems to be reduced in individuals with autism (Fitzpatrick et al., 2013, 2016; Marsh et al., 2013). Conversely, the instruction to (intentionally) synchronize movements provides an explicit trigger for movement interaction, and may be expected to yield higher degrees of synchronization in individuals with autism, who are known to thrive on explicit instructions (Schwarzkopf et al., 2014).

Empirically, these predictions can be tested in persons (in dyads) who move one of their limbs rhythmically but, initially, at slightly different tempi and/or phasing. Once they see each other’s movements (Richardson et al., 2007; Varlet et al., 2012) interpersonal interactions are expected to induce entrainment. To address the degree of social initiative, participants can be instructed to either continue moving at the initial tempo and/or phasing (unintentional condition: no social initiative required) or to synchronize the movements with the partner (intentional condition: social initiative required). Less entrainment is expected for autism–TD dyads than for TD–TD dyads in the unintentional condition (Fitzpatrick et al., 2013, 2016; Marsh et al., 2013), but not necessarily in the intentional condition, given the explicit instruction to produce synchronization (but see also Fitzpatrick et al., 2016). Moreover, by analyzing the adaptations in the individual movement patterns during the first instances of entrainment, the degree to which participants with autism demonstrate social initiative can be further examined.

### Reciprocity Of Mutual Adjustments

Autism is not only characterized by a reduced tendency to initiate social interactions, but also by reduced reciprocity during social interactions, which affects the dynamics of the ongoing adjustments between the interactants. Measuring such reciprocity requires a technique to disentangle the dynamic contributions of each participant to the reciprocal interaction. Indeed, recent tests for reciprocal behavior in autism (Backer van Ommeren et al., 2015) demonstrated that such a dynamic approach yields an IQ-independent assessment. Individuals with autism show clear limitations to reciprocate during an interaction process with another person, although initial evidence suggest improvement when appropriate support is provided (Holt and Yuill, 2014). However, targeting the mutual adjustments during the interaction requires more detailed analyses of behavior, taking into account the ongoing contribution of each interactant in real time.

If bodily connectedness is a marker for autism, asymmetries are expected in the movement interactions between individuals. When two individuals synchronize their movements, the degree to which they contribute to the joint coordination pattern may differ. Whereas a person with autism may be expected to adapt his/her movements less to those of the partner, it is possible that this tendency is (partly) compensated by enhanced adaptations by the partner, thereby potentially obscuring the reduced bodily connectedness in the person with autism. Conversely, it is also possible that the partner shows less bodily connectedness when coordinating with an individual with autism. It is therefore important to establish the extent to which each person adapts his/her movements to those of the partner (Oullier et al., 2008). This can be done, for example, in the entrainment experiment described in the previous section by determining how much the phase and/or frequency of each person’s movements, due to the mutual interactions, deviates from the initial values. The same can be done for a more challenging coordination task like the ‘mirror game,’ in which dyads are instructed to make creative yet synchronized rhythmic movements. For this paradigm, Słowiński et al. (2016) recently developed a technique to determine the degree of movement adaptation, based on observed deviations of the ‘individual motor signatures.’

A potentially stronger test involves the application of brief, unexpected perturbations that disrupt the interpersonal coordination pattern through a temporary arrest of one of the limbs (Peper et al., 2013). To re-establish the original coordination pattern, at least one of the persons has to adapt the phasing of his/her movements. In TD–TD dyads that intentionally synchronize their movements, both persons contribute approximately equally to this return process, yielding an adaptation ratio of about 0.5 (Peper et al., 2013). Participants with autism may show reduced adaptions of their movement phasing to the perturbed movements of the partner, resulting in a lower adaptation ratio and longer adaptation time before the original pattern is re-established.

So far, this technique has only been applied to situations in which TD–TD dyads were instructed to synchronize their movements. However, given the reduced social initiative in autism, it seems worthwhile to examine asymmetries in reciprocity during spontaneous entrainment (no instruction with respect to interpersonal coordination) as well. Since perturbation tests require a more advanced set-up than an entrainment test, it is useful to compare the results for both paradigms to determine whether the entrainment paradigm would suffice in this regard.

## Conclusion

To date, most research on the defining deficits of autism in social interactions has focused on social communicative behavior or cognition. Although the role of underlying bodily movement has largely been neglected, perceptuo-motor impairment (Spencer et al., 2000; Gepner and Mestre, 2002) may be expected to affect socio-cognitive functioning (Leary and Hill, 1996; De Jaegher, 2013; Cook, 2016). By focusing on covert movement coordination characteristics, the influences of acquired social or cognitive skills can be circumvented, uncovering the ways in which autism may be associated with impaired bodily connectedness (Marsh et al., 2013). The coordination dynamics approach offers experimental paradigms for scrutinizing specific aspects of bodily connectedness, which may help to assess defining characteristics of autism, such as poor social initiative and reciprocity. To enhance the sensitivity of the proposed empirical methods additional modulations of the social setting may be applied, such as implicit social priming (Raffard et al., 2015).

If these assessments are successful, follow-up research may address their potential application in diagnostic procedures, for instance, by developing affordable set-ups (e.g., registration with Microsoft Kinect; Clark et al., 2013), determining whether human partners can be replaced by virtual partners/robots with (Dumas et al., 2014; Słowiński et al., 2016) or without (Meerhoff et al., 2014; Zhao et al., 2015) interactional simulation software, and defining simplified protocols suitable for clinical use. Thus, a focus on bodily connectedness may contribute to the development of assessment tools that are sensitive to the ongoing dynamics of social initiative and reciprocity in interpersonal interactions, while bypassing cognitive compensation strategies. In addition, it would provide additional fuel for theoretical considerations, regarding the underlying causes of autism and their potential relation to motoric and/or perceptual problems as highlighted by the embodied cognition account (von Hofsten and Rosander, 2012; De Jaegher, 2013).

## Author Contributions

CP and SB: conception and writing of article; SW: critical reading and writing of article.

## Conflict of Interest Statement

The authors declare that the research was conducted in the absence of any commercial or financial relationships that could be construed as a potential conflict of interest.
